# Child Opportunity Index and Access to Firearms Among Adolescents and Young Adults

**DOI:** 10.1001/jamahealthforum.2025.3910

**Published:** 2025-09-19

**Authors:** Nicole Koepke, Warren D. Frankenberger, Katelin Hoskins, Darcy L. Brodecki, Walter Faig, Anireddy R. Reddy

**Affiliations:** 1 Center for Pediatric Nursing Research and Evidence-Based Practice, Children’s Hospital of Philadelphia, Philadelphia, Pennsylvania; 2 School of Nursing, University of Pennsylvania, Philadelphia; 3 Leonard Davis Institute of Health Economics, University of Pennsylvania, Philadelphia; 4 Children’s Hospital of Philadelphia Research Institute, Philadelphia, Pennsylvania; 5 Department of Anesthesiology and Critical Care Medicine, Children’s Hospital of Philadelphia, University of Pennsylvania Perelman School of Medicine, Philadelphia; 6 PolicyLab, Center for Violence Prevention, Children’s Hospital of Philadelphia, Philadelphia, Pennsylvania

## Abstract

This cohort study investigates the association between the Child Opportunity Index and adolescent and young adult firearm access across a large pediatric primary care network in the US.

## Introduction

Firearm injury is the leading cause of adolescent morbidity and mortality in the US.^1^ Social determinants of health, including poverty, neighborhood deprivation, and low child opportunity, are associated with pediatric firearm injury.^2,3^ However, it is unclear if child opportunity is associated with adolescents’ immediate access to firearms, which is critical for informing injury prevention efforts. Across a large pediatric primary care network, we investigated the association between Child Opportunity Index (COI) and adolescent and young adult firearm access, hypothesizing that lower COI would be associated with higher immediate access to firearms.

## Methods

This was a single-center, retrospective cohort study of adolescents and young adults (aged 13-21 years) who presented to a well visit between January 1 and December 31, 2022, in the Children’s Hospital of Philadelphia primary care network (6 urban and 26 suburban sites). Each patient was assigned an Adolescent Health Questionnaire that included 2 firearm access questions: (1) “Is there a gun in your home or brought into your home at any point?” and (2) “If you wanted, could you obtain a gun within a day?” We classified immediate access as in-home access (yes to question 1 or both questions) or outside-home access (no to question 1 and yes to question 2). We stratified results by nationally normed COI 2.0 quintiles based on patients’ home census tract; COI 2.0 is an area-based index of 29 indicators across education, health/environment, and socioeconomic domains.^4^ The adjusted odds of immediate firearm access (adjusted for age, sex, and payer) by COI quintile were calculated using multivariable logistic regression.

We adhered to STROBE guidelines for reporting observational studies. This study was deemed exempt by the institutional review board at the Children’s Hospital of Philadelphia because it was retrospective analysis of deidentified data. Data were analyzed from May to December 2024.

## Results

Of 58 495 total well visits during the study period, 54 396 individual patients completed the Adolescent Health Questionnaire (93% completion rate). Of these patients, 53 926 had sufficient address data to determine COI (<1% missingness; Table), which were used for complete case analysis. A total of 13.9% of patients reported in-home access, and 1.3% reported outside-home access to firearms. Additionally, 4.4% of these patients identified as Asian, 25.5% as Black or African American, 8.3% as Hispanic, 54.1% as White, 16.1% as another race, and less than 1% as another ethnicity. While 20.4% of patients were from urban areas, 79.6% were from suburban areas. The distribution by COI was 18.2% very low, 6.7% low, 9.2% moderate, 18.7% high, and 47.2% very high. Compared to those in very low COI neighborhoods, patients in moderate, high, and very high quintiles had higher odds of in-home access and lower odds of outside-home access (Figure).

**Table.** Characteristics of Respondents by Child Opportunity Index Quintile

Characteristic	No. (%)					
	Very low	Low	Moderate	High	Very high	Total
Respondents	9793 (18.2)	3602 (6.7)	4982 (9.2)	10 111 (18.7)	25 438 (47.2)	53 926 (100)
Age, y						
13-18	8600 (87.8)	3107 (86.3)	4283 (86.0)	8912 (88.1)	22 079 (86.8)	46 981 (87.1)
18-21	1193 (12.2)	495 (13.7)	699 (14.0)	1199 (11.9)	3359 (13.2)	6945 (12.9)
Missing	0	0	0	0	0	0
Sex						
Female	5044 (51.5)	1822 (50.6)	2522 (50.6)	5006 (49.5)	12 533 (49.3)	26 927 (49.9)
Male	4748 (48.5)	1780 (49.4)	2458 (49.3)	5103 (50.5)	12 902 (50.7)	26 991 (50.1)
Missing	1 (<1)	0	2 (<1)	2 (<1)	3 (<1)	8 (<1)
Ethnicity						
Hispanic	853 (8.7)	484 (13.4)	651 (13.1)	1016 (10.0)	1477 (5.8)	4481 (8.3)
Non-Hispanic	8906 (90.9)	3094 (85.9)	4299 (86.3)	9020 (89.2)	23 808 (93.6)	49 127 (91.1)
Other^a^	33 (<1)	24 (<1)	32 (<1)	74 (<1)	152 (<1)	315 (<1)
Missing	1 (<1)	0	0	1 (<1)	1 (<1)	3 (<1)
Race						
Asian	200 (2.0)	136 (3.8)	137 (2.7)	295 (2.9)	1581 (6.2)	2349 (4.4)
Black or African American	7967 (81.4)	1734 (48.1)	1321 (26.5)	1263 (12.5)	1488 (5.8)	13 773 (25.5)
White	505 (5.2)	1040 (28.9)	2500 (50.2)	6877 (68.0)	18 226 (71.6)	29 148 (54.1)
Other^b^	1121 (11.4)	692 (19.2)	1024 (20.6)	1676 (16.6)	4143 (16.3)	8656 (16.1)
Missing	0	0	0	0	0	0
Payer						
Private	3300 (33.7)	1842 (51.1)	3129 (62.8)	7594 (75.1)	21 818 (85.8)	37 383 (69.3)
Public	6791 (69.3)	1759 (48.8)	1849 (37.1)	2507 (24.8)	3603 (14.2)	16 509 (30.6)
Missing	2 (<1)	1 (<1)	4 (<1)	10 (<1)	17 (<1)	34 (<1)
Urban or suburban area						
Urban	7667 (78.3)	1291 (35.8)	1040 (20.9)	786 (7.8)	229 (<1)	11 013 (20.4)
Suburban	2126 (21.7)	2311 (64.2)	3942 (79.1)	9325 (92.2)	25 209 (99.1)	42 913 (79.6)
Missing	0	0	0	0	0	0
Firearm access						
No immediate access	8610 (87.9)	3128 (86.8)	4226 (84.8)	8087 (80.0)	21 700 (85.3)	45 751 (84.8)
Immediate in-home access	978 (10.0)	416 (11.5)	688 (13.8)	1925 (19.0)	3471 (13.6)	7478 (13.9)
Immediate outside-home access	205 (2.1)	58 (1.6)	68 (1.4)	99 (1.0)	267 (1.0)	697 (1.3)
Missing	0	0	0	0	0	0

^a^ The other ethnicity category is inclusive of those reporting other ethnicity and refused, unknown, or choose not to disclose. These were combined due to small sample size.

^b^ The other race category is inclusive of those reporting American Indian or Alaska Native, Indian, Native Hawaiian or Other Pacific Islander, more than 1 race, other race, and refused, unknown, or choose not to disclose. These were combined due to small sample size.

**Figure.**
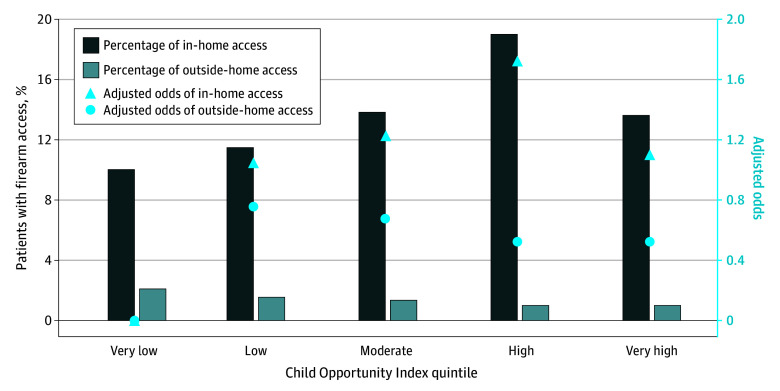
Immediate Firearm Access by Child Opportunity Index Quintile Odds are adjusted for age, sex, and payer.

## Discussion

This cohort study surveying adolescents and young adults across a diverse pediatric primary care network found associations between COI and immediate firearm access. As hypothesized, patients in the very low COI quintile had higher odds of outside-home access to firearms; however, we were surprised to find the inverse association for in-home access. While overall in-home access was 13.9%, the rates of access differed by COI, with almost 1 in 5 patients in high COI areas reporting in-home access. Outside-home access was low overall (1.3%) but predominantly in very low and low COI areas. The relationship between COI and type of immediate firearm access could inform intervention. Evidence-based secure firearm storage counseling and locking device distribution,^5^ for example, may be more impactful in COI areas with higher in-home access; conversely, community-engaged programming or neighborhood-level interventions such as abandoned home remediation^6^ may be more appropriate in lower COI neighborhoods. The strengths of this study include large sample size, high response rate, and inclusion of patients in both urban and suburban areas. However, the epidemiology of firearm access and injury is affected by geography, policy, and other factors that limit generalizability. Further research is needed to assess the relationship between COI and firearm access, as well as how best to target interventions to reduce adolescent and young adult firearm injury.

## Notes

Supplement.
Data Sharing Statement
