# Supplementary material for: Child Opportunity Index and Access to Firearms Among Adolescents and Young Adults
Source: JAMA Health Forum. 2025 Sep 19;6(9):e253910. doi: 10.1001/jamahealthforum.2025.3910 (PMC12449723; doi:10.1001/jamahealthforum.2025.3910)
Supplement: Supplement. — Data Sharing Statement [file jamahealthforum-e253910-s001.pdf]

Data Sharing Statement

Koepke. Child Opportunity Index and Access to Firearms Among Adolescents and Young Adults. *JAMA Health Forum*. Published September 19, 2025.
doi:10.1001/jamahealthforum.2025.3910

Data
Data available: No
